# The Role of Hypoxia and the Immune System in Tumor Radioresistance

**DOI:** 10.3390/cancers11101555

**Published:** 2019-10-14

**Authors:** Paul N. Span, Jan Bussink

**Affiliations:** Radiotherapy and OncoImmunology Laboratory, Department of Radiation Oncology, Radboud University Medical Center, 6500 HB Nijmegen, The Netherlands

Radiotherapy is given to a majority of patients with cancer, and remains one of the most (cost)effective treatment options available. However, several mechanisms attenuating the efficacy of irradiation have long been known to occur, such as hypoxia, DNA damage repair, repopulation, cell cycle redistribution and, more recently, immunological responses. Radiobiology research into tumor radioresistance has thus focused on targeting these mechanisms to increase radiotherapy effectiveness by, e.g., hypoxia modification, or using combination therapy with DNA damage repair or growth factor receptor inhibitors and reducing the overall treatment time. The impact of some of these classic mechanisms on tumor radioresistance has changed, however, with the widespread use of novel radiotherapy regimens such as heavy ions/particles, stereotactic radiotherapy, radiotherapy and immunotherapy combination, etc. Additionally, recent insights in additional mechanisms such as stem cells/stemness, tumor metabolism, and the effect of irradiation on the immune system has led to rapid development of additional targets for combination therapy with irradiation.

In this Special Issue of *Cancers*, several studies have been published addressing the current state of affairs and developing novel aspects of tumor radioresistance, with a total of five original data papers and three reviews.

One of the most well-studied causes for radioresistance in solid tumors is hypoxia [1], as also evidenced by the Nobel Prize in Physiology or Medicine 2019 being awarded to William G. Kaelin Jr., Sir Peter J. Ratcliffe and Gregg L. Semenza “for their discoveries of how cells sense and adapt to oxygen availability”. 

In [2], van der Heijden and colleagues compared several hypoxia profiles to acute and chronic hypoxia profiles obtained in vitro. Somewhat surprisingly, acute hypoxia profiles showed a stronger association with patient outcome than chronic hypoxia profiles. Thus, Darwinian selection of hypoxia-tolerant apoptosis-resistant cells is unlikely to be a mechanism leading to radioresistance. Most tumor cells are able to survive and even proliferate during short episodes of hypoxia, during which they are also resistant to DNA damage from irradiation. 

This tolerance for hypoxia is also transmitted from one tumor cell to distant tumor cells by means of extracellular vesicles, as reviewed by Zonneveld et al. [3]. These vesicles contain factors such as proteins, mRNAs and micro-RNAs that impact hypoxia tolerance mechanisms such as the HIF1 pathway, the Unfolded Protein Response (UPR) and autophagy. 

A gene that is induced by hypoxia via the PERK-AT4 arm of the UPR and is associated with hypoxia tolerance [4] is Tribbles-3 (TRIB3). In this issue, Lee et al. found that TRIB3 is directly involved in radiosensitivity via Notch signaling [5]. Knockdown of TRIB3 in radioresistant breast cancer cells led to an increase in radiosensitivity, together with a decrease in CD24^−^/CD44⁺ cancer stem cell (CSC) population. 

This CSC population plays an important role in radioresistance as reviewed in this issue by Schulz et al. [6]. CSCs are inherently radioresistant through an increased DNA repair capacity, and given their repopulation potential an important target for radiotherapy. The CSCs are also hypoxia-tolerant, and as such are considered to reside in the so-called “hypoxic niche”, which adds to their radioresistant phenotype.

Wang et al. [7] showed that the Hedgehog pathway is involved in cancer cell stemness and treatment resistance. Moreover, targeting this pathway in esophageal cancer using vismodegib led to a decrease in cancer stemness, and not only sensitized for radiation but also for carboplatin.

The role of the immune system in anti-tumor responses is becoming clearer, and especially its interaction with other treatment modalities such as radiotherapy. Wu et al. [8] found that castration-resistant prostate cancers (CRCP) exhibit an immunosuppressive tumor microenvironment. Androgen deprivation therapy enhanced the effects of radiotherapy in CRCP by changing this microenvironment, leading to less myeloid-derived suppressor cells and more tumor-infiltrating T-cells. This study emphasizes the role of the tumor microenvironment in the efficacy of radiotherapy.

Another component of the tumor microenvironment that influences radiosensitivity through immunomodulation is cancer-associated fibroblasts (CAFs). Berzaghi et al. [9] found that CAFs modulate the function of macrophages, and that this effect does not change after single-dose or fractionated radiotherapy.

The anti-tumor immune response that is induced by radiotherapy is the subject of the review by Boustani et al. [10]. Combining radiotherapy with immune therapy using immune checkpoint inhibitors and/or targeting the cGAS-STING pathway is likely to enhance the efficacy of radiotherapy, as well as the possibility of systemic (abscopal) effects.

Over 120 years after the first use of radiation in the treatment of cancer, radiotherapy continues to be an essential curative and palliative treatment option for the majority of cancer patients. This Special Issue describes several aspects of radioresistance, from hypoxia to the interaction with the immune system. It will be exciting to see how the research fields of radiobiology and tumor immunology might together contribute to the prevention of radioresistance and harness the anti-tumor immune response for future treatment options involving radiotherapy.

## References

[1] Span P.N., Bussink J. (2015). Biology of hypoxia. Semin. Nucl. Med..

[2] Van der Heijden M., de Jong M.C., Verhagen C.V.M., de Roest R.H., Sanduleanu S., Hoebers F., Leemans C.R., Brakenhoff R.H., Vens C., Verheij M. (2019). Acute Hypoxia Profile is a Stronger Prognostic Factor than Chronic Hypoxia in Advanced Stage Head and Neck Cancer Patients. Cancers (Basel).

[3] Zonneveld M.I., Keulers T.G.H., Rouschop K.M.A. (2019). Extracellular Vesicles as Transmitters of Hypoxia Tolerance in Solid Cancers. Cancers (Basel).

[4] Wennemers M., Bussink J., Scheijen B., Nagtegaal I.D., van Laarhoven H.W., Raleigh J.A., Varia M.A., Heuvel J.J., Rouschop K.M., Sweep F.C. (2011). Tribbles homolog 3 denotes a poor prognosis in breast cancer and is involved in hypoxia response. Breast Cancer Res..

[5] Lee Y.C., Wang W.L., Chang W.C., Huang Y.H., Hong G.C., Wang H.L., Chou Y.H., Tseng H.C., Lee H.T., Li S.T. (2019). Tribbles Homolog 3 Involved in Radiation Response of Triple Negative Breast Cancer Cells by Regulating Notch1 Activation. Cancers (Basel).

[6] Schulz A., Meyer F., Dubrovska A., Borgmann K. (2019). Cancer Stem Cells and Radioresistance: DNA Repair and Beyond. Cancers (Basel).

[7] Wang D., Nagle P.W., Wang H.H., Smit J.K., Faber H., Baanstra M., Karrenbeld A., Chiu R.K., Plukker J.T.M., Coppes R.P. (2019). Hedgehog Pathway as a Potential Intervention Target in Esophageal Cancer. Cancers (Basel).

[8] Wu C.T., Chen W.C., Chen M.F. (2018). The Response of Prostate Cancer to Androgen Deprivation and Irradiation Due to Immune Modulation. Cancers (Basel).

[9] Berzaghi R., Ahktar M.A., Islam A., Pedersen B.D., Hellevik T., Martinez-Zubiaurre I. (2019). Fibroblast-Mediated Immunoregulation of Macrophage Function Is Maintained after Irradiation. Cancers (Basel).

[10] Boustani J., Grapin M., Laurent P.A., Apetoh L., Mirjolet C. (2019). The 6th R of Radiobiology: Reactivation of Anti-Tumor Immune Response. Cancers (Basel).

